# DaiCee: A database for anti-cancer compounds with targets and side effect profiles 

**DOI:** 10.6026/97320630016843

**Published:** 2020-11-30

**Authors:** Manikkam Rajalakshmi, Sugumaran Suveena, Periyasamy Vijayalakshmia, Sabapathy Indu, Anita Roy, Antony Ludas

**Affiliations:** 1 DBT-BIF Centre, PG & Research Department of Biotechnology & Bioinformatics, Holy Cross College (Autonomous) (Affiliated to Bharathidasan University), Trichy, Tamilnadu, India

**Keywords:** Drug, properties, SMILES, ADME/T, target

## Abstract

Identification of the toxicity of compounds is more crucial before entering clinical trials. Awareness of physiochemical properties, possible targets and side effects has become a major public health issue to reduce risks. Experimental determination of analyzing the physiochemical properties of a drug, their interaction with specific receptors and identifying their side-effects remain challenging is time consuming and costly. We describe a manually compiled database named DaiCee database, which contains 2100 anticancer drugs with information on their physiochemical properties, targets of action and side effects. It includes both synthetic and herbal anti-cancer compounds. It allows the search for SMILES notation, Lipinski's and ADME/T properties, targets and side effect profiles of the drugs. This helps to identify drugs with effective anticancer properties, their toxic nature, drug-likeness for in-vitro and in-vivo experiments. It also used for comparative analysis and screening of effective anticancer drugs using available data for compounds in the database. The database will be updated regularly to provide the users with latest information. The database is available at the URL http://www.hccbif.org/usersearch.php

## Background

**Figure 1:**
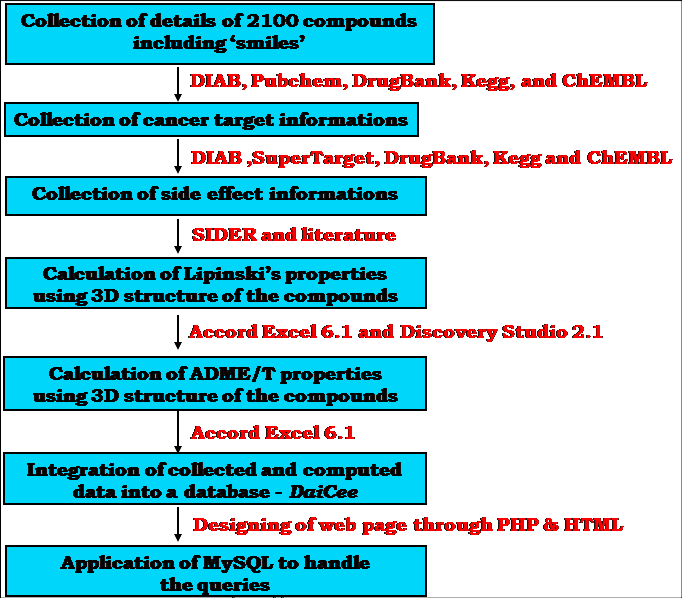
Flowchart for database creation

Cancer is that the second leading explanation for death worldwide. As multiple targets are often involved in cancer disease, much attention has been paid to search drugs involving many targets [1-3]. Knowledge of protein targets of drugs is useful in solving various problems in drug discovery process [4,5]. Only a limited number of targets are identified as related to approved drugs so far [4,6,7]. The therapeutic goals of a variety of medicinal drugs are still unclear. Experiment for identifying target for a drug molecule is time-consuming, expensive, and limited in small-scale research, computational methods are needed to decrease time and costs for drug discovery [8-11]. Computational methods can provide supporting evidences to the drug target experiments and accelerate the drug discovery [12]. Therefore it is of interest to describe DaiCee, a Database for anti-cancer compounds with targets and side effect profiles for further evaluation using in vitro and in vivo assays. The flow chart of entire work was given in Figure 1.

## Methodology:

### Data collection:

The database includes 224 anticancer compounds. Its targets and the side effect profile were collected from scientific literatures, various databases, such as DIAB [13], Kegg [14], Supertarget [15], Pubchem [16], ChEMBL [17], Drug Bank [18] and SIDER [19]. The database was organized in the alphabetical order of the compounds that simplifies the process of finding the target, properties and side effect profiles. The dataset of the compound were prepared for property prediction.

### Generating the properties:

Properties of each compound such as Lipinski's rule of five, ADMET were calculated using Accord Excel 6.1 version. Accord Excel did not provide exact molecular weight and LogP value, rather it gave,if molecular weight less than 500, the result as False, otherwise True. If logP value is less than 5, the result will be "False" otherwise "True". So in order to overcome this difficulty we used Discovery studio 2.1 version, with the help of which we calculated the value of molecular weight and LogP. Accelrys Discovery Studio (2.1) is a life science modeling and simulation suite of application focused on optimizing the drug discovery process. It makes easier to examine the properties of large and small molecules. Accord for Excel allows scientists to display chemical structures and reactions, perform chemical calculations, analyze R-groups, and query by substructure or similarity directly within Excel.

### Database design:

The database is developed as a result of integration of all the collected and computed data into a single storehouse through PHP and HTML as front end and MySQL as back end.

### Features of the new database:

The database includes compound name, SMILES, drug likeness properties, target and side effect profile as separate fields. Advanced search links were provided to access through compound name, SMILES, target name, molecular formula or molecular weight. This is open source and freely available.

## Results and Discussion:

**Figure 2:**
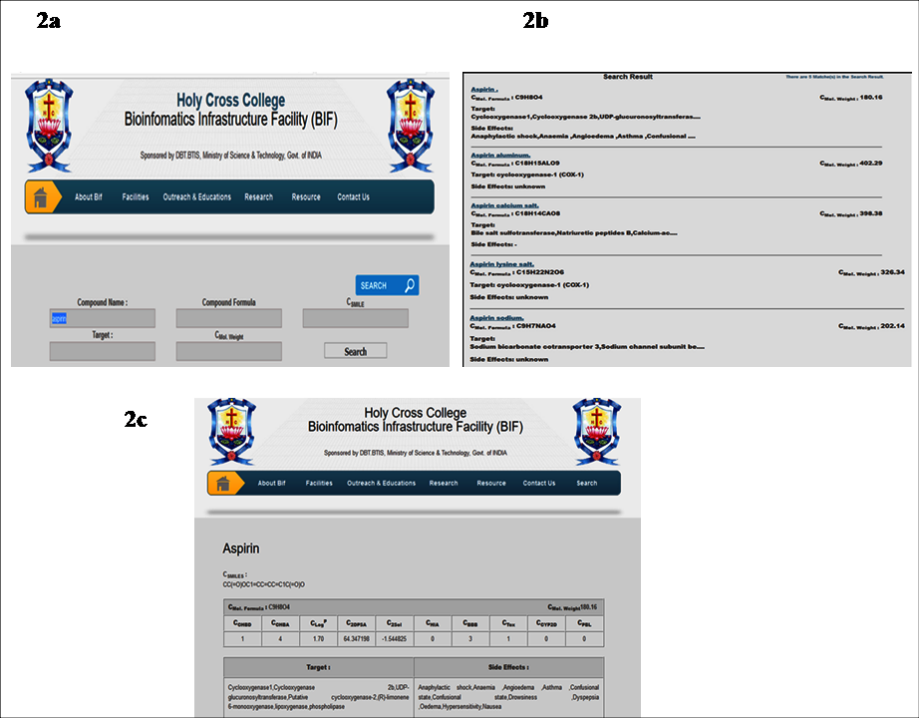
Web Interface for the database (a) Submission page of Daicee Database; (b) A sample of data retrieved for Aspirin from Daicee database; (c) ADME/T properties of the Aspirin retrieved from Daicee database

The compound details in the database can be accessed by text search by providing details such as compound name or Protein target details or compound formula or molecular weight of the compound or Smile notation of the compound (Figure 2a). Here, Compound name"Aspirin" has given for submission as an example. The results of Aspirin obtained from the database contains all compounds related to Aspirin in the database such as Aspirin aluminum, Aspirin calcium salt, Aspirin lysine salt and Aspirin sodium along with Aspirin are generated (Figure 2b). The main page for each entry provides the following information: Molecular formula, Lipinski's rule of five, ADME/T properties, Protein targetand side effect profile (Figure 2c).

Drug target discovery, which aims to rapidly and accurately identify drug targets with true potential, is a crucial step in the discovery process and also plays a vital role in new therapeutics. In pharmaceutics, drugs generally fail in the clinic for two reasons: they either do not work or are proved to be unsafe. Drug target validation and identification of undesirable effects are among the main challenges in developing new drugs. A database with collection of a set of information on drugs available for a particular disease on in general would help the researchers for the easy search and knowledge on the compounds, help to guide and speed up the laborious and costly experimental determination on screening or identification of the potential biological activity of the drug. Similar to Jayashreeet al. 2010 & John et al., 2012 [20] the Daicee database is also enriched by user-friendly interface, important keywords are hyperlinked and pictorial representations. DaiCee database stands unique with information on both anticancer agents from different sources, not particular in its source. The ADME/T and Lipinski rule of five provided in the database was identified for those drugs with no data reported on it. The information on the side-effect profiles of the anticancer drugs of natural as well as synthetic has been found to be a distinctive parameter in DaiCee, which was lacking in other reported databases. It also contains some novel compounds having anticancer effect. Daicee is a unique knowledge and analysis environment for small molecules. It provides calculated molecular descriptors, experimental assay results as target, and literature-based information side effects, allowing integration of both new and established information in a single, public resource.

## Conclusion

We describe a manually compiled database named DaiCee database, which contains 2100 anticancer drugs with information on their physiochemical properties, targets of action and side effects. It includes both synthetic and herbal anti-cancer compounds. It allows the search for SMILES notation, Lipinski's and ADME/T properties, targets and side effect profiles of the drugs. This helps to identify drugs with effective anticancer properties, their toxic nature, and drug-likeness for in-vitro and in-vivo experiments.
